# Impact of Race, Socioeconomic Status, and Geography on Healthcare Outcomes for Children With Sickle Cell Disease in the United States: A Scoping Review

**DOI:** 10.7759/cureus.56089

**Published:** 2024-03-13

**Authors:** Sameerah Wahab, Kaylan Kelly, Mariah Klingler, Annalena Pirovic, Katerina Futch, Christopher Rennie, Devon Durham, Donna Herber, Grant Gramling, Shawn Price, Joshua M Costin

**Affiliations:** 1 Osteopathic Medicine, Nova Southeastern University Dr. Kiran C. Patel College of Osteopathic Medicine, Tampa, USA; 2 Osteopathic Medicine, Nova Southeastern University Dr. Kiran C. Patel College of Osteopathic Medicine, Davie, USA; 3 Medical Education, Nova Southeastern University Dr. Kiran C. Patel College of Allopathic Medicine, Fort Lauderdale, USA

**Keywords:** pediatric population, access to healthcare and health outcomes of vulnerable populations, healthcare outcomes, cultural identity, geographical factors, socioeconomic status (ses), race inequities, sick sickle cell disease (scd)

## Abstract

A large proportion of patients with sickle cell disease (SCD) identify as Black or African American (AA). Social bias and stigma in healthcare outcomes for children with SCD are impossible to explore without considering the impact of racial/cultural identity, socioeconomic status (SES), and geography. It is important to understand the current influences of social movements, expanded health insurance coverage, and telehealth on these variables when considering healthcare outcomes for patients with SCD. The objective of this study was to determine the roles of racial identity, SES, and geography in healthcare outcomes for the pediatric population of children with SCD in the United States (US). This study is a scoping review following Preferred Reporting Items for Systematic Reviews and Meta-Analyses (PRISMA) guidelines. The databases utilized included Cochrane, CINHAL, Medline, and Nursing and Allied Health Collection, all accessed through the EBSCO Information Services. Studies met the following inclusion criteria: published in English, pediatric patients residing in the US, and published between 2017 and 2022. Search terms included “sickle cell” AND “pediatric”, which were then combined with “minority” OR “racial” OR “rural” OR “urban” OR “poverty” OR “income” OR "socioeconomic status”. The initial search yielded 635 unique articles, with 17 articles meeting full inclusion criteria. Overall, it was clear that there are examples of positive effects of race, low SES, and rural geographic location on positive health outcomes, though a large number of studies oscillated between showing negative associations or no association at all. Barriers to care for patients with SCD are multifaceted, making it difficult to isolate and analyze the impact of individual variables. Many studies demonstrated the significance of family, community, and institutional relationships as positive support for patients with SCD. This review highlights the need for additional research on the healthcare outcome benefits of patient/familial support groups aiming to bring together patients who share racial experience and SCD diagnosis regardless of SES and geography.

## Introduction and background

Sickle cell disease (SCD) is an inherited blood disorder that is caused by a mutation in the β-globin gene of hemoglobin, allowing tetramer formation with 𝛼-globin and creating sickle hemoglobin (HbS) [1]. The altered shape of HbS causes the adhesion of red blood cells to the walls of blood vessels, leading to vascular complications and acute pain in patients with SCD [1]. Within the United States (US), SCD is prevalent primarily among African American (AA) communities. According to the Centers for Disease Control and Prevention (CDC), approximately 100,000 Americans are living with SCD, and one in 13 AA babies is born with sickle cell trait (SCT) [2]. SCD was historically thought to be a childhood disease; however, the life expectancy of SCD patients has increased, shifting it to a chronic disease in adults due to early treatment of infection, newer immunizations, and universal newborn screening [3]. Despite these advancements, the average life expectancy of individuals with SCD is 30 years less than the general population [4]. In the early pediatric years, providing access to multifactorial support from diagnosis to treatment has the potential to extend and improve the quality of life for patients. Areas of healthcare fallout that may impact an individual's accessibility to prompt, appropriate, and continuous care include racial/cultural identity, socioeconomic status (SES), and geography.

In the US, the incidence of SCD is more than double that of other genetic conditions, such as cystic fibrosis (CF) and hemophilia, whose prevalence disproportionately affects the White population and is rare in the Black population. However, comparatively speaking, the availability of SCD-specific treatment centers remains markedly scarcer [4]. Patients with CF and hemophilia benefit from more than 130 comprehensive treatment centers nationwide, whereas patients suffering from SCD, especially those in low-income urban and rural communities, may struggle to access basic general care, leaving potential reliance on emergency departments (ED). At the same time, the care provided is not free of mismanagement. In a 2013 study, AA patients with SCD waited 25% longer than the general patient sample; when controlled for race and triage priority, patients with SCD experienced wait times 50% longer than the general population [5]. While in the ED, patients with SCD often experience additional forms of discrimination such as name-calling by staff (associated with a negative perception of those with SCD), attributable to medical professionals’ lack of comprehension of the complexity of patients’ needs for pain management [6]. Disease ignorance and bias prevent these patients from receiving timely care and damage the patient-provider relationship, which can be considered the foundation of healthcare [7]. In recent years, it is unclear how the changing social climate has impacted such issues.

SES appears to be an additional barrier of interest. The steady advancement of science is not always reflected in public health, as economic disparities often discourage access to healthcare [8]. According to the US Census, AAs had the highest poverty rate of all races, representing 23.8% of the poverty population [9]. When examining various SES factors in pediatric healthcare in previous literature, it has been previously reported that the lowest-income and least-educated groups experienced lower levels of health compared to those with the advantage of higher SES [10]. With difficulty accessing care due to SES-related constraints, these patients may often rely on EDs for the management of disease crises, especially if utilizing public insurance, such as Medicare or Medicaid [4,11,12]. Of note, high copayments may discourage care altogether [12]. However, even when controlling for insurance, income disparities, and unmet health needs-disparities were still found between low- and high-income families [13]. Patients with healthcare coverage still experience gaps in service exacerbated by lower SES [12]. Lower SES typically encompasses poverty and malnutrition, putting these patients at a unique risk when factoring in food insecurity. A study done by Prussien et al. reported that complex socioeconomic and environmental influences related to patients with SCD showed significant cognitive deficits relative to the normative mean, matched siblings, and healthy controls [14]. Even in the absence of SCD-related cerebral infarction, it has been noted these patients still show deficits, but it is unclear if the cause may be indirect effects of their illness or complex social factors [15]. This emphasizes the importance of the quality of early education for children with SCD, which can be disrupted by underserved school districts in low-income areas.

Within the US, an individual’s geographical location serves as a major potential barrier to healthcare that can significantly impact patients with SCD. Disparities in healthcare access and outcomes occur most frequently among inner-city and rural poor, with differences in urban and rural facilitators and barriers to health [16]. This trend stems from numerous factors, including a lack of health services and substandard infrastructure, as well as difficulty in attracting and retaining medical professionals in rural areas [17]. Beyond the services themselves, there is an inherent disadvantage in rural areas due to accessibility such as insufficient public transport, scarce broadband internet services, and subpar educational opportunities [17]. As a result, studies have shown that among pediatric patients, Black children specifically have a disproportionately higher mortality rate in these rural areas [18].

As discussed above, there exists a unique interplay between race, SES, and geography on the overall outcome of care in patients with SCD. In recent years, the conversation about race in healthcare has come to the forefront as the Black Lives Matter movement attracted attention and support. Social bias and stigma in healthcare are almost impossible to explore without considering the impact of racial/cultural identity, SES, and geography. Therefore, a scoping review was conducted to address the unique challenges of care delivery to pediatric patients with SCD in the US and identify gaps in the literature regarding this subject.

## Review

Materials and methods

The Preferred Reporting Items for Systematic Reviews and Meta-Analyses (PRISMA) guidelines were strictly followed over the course of this review. All inclusion criteria were decided prior to beginning the study.

Eligibility Criteria

To meet inclusion criteria, articles had to be written in English and take place in the US. Studies need to have been published between 2017 and 2022 in a peer-reviewed journal and could be experimental, quasi-experimental, or descriptive studies, as well as case reports and case series. The article had to focus on the pediatric population with SCD and any of the following: stigma and bias in care, healthcare inequity/disparity, geographic location, racial/ethnic identity, family income, healthcare accessibility and social services, pain management, age at diagnosis, SES, mortality rate, life expectancy, and hospital or emergency department utilization.

Search Strategy

Three authors (MK, KF, and DH) created a search strategy using four databases: Cochrane, Cumulative Index to Nursing and Allied Health Literature (CINHAL), Medline, and Nursing and Allied Health Collection. All databases were included in a single search through EBSCOhost (EBSCO Information Services) on October 5, 2022. All authors reviewed the unique studies, and 80% of the authors had to mark an article as relevant to the study and meeting the inclusion criteria to be retained for inclusion in this study. The screening and selection process was documented using a PRISMA flowchart.

The research question was based on the population, concept, and context (PCC) strategy: P for pediatric patients aged 0-18 with sickle cell diagnosis; C for key aspects of care delivery (effects of race, SES, and geography); and C, within the US medical care accessibility based on SES and race. Three authors (DH, MK, KF) were responsible for conducting the searches independently and used “Sickle cell” AND “pediatric OR child OR children OR adolescent OR infant” as the initial search, then adding in the additional terms “rural” OR “urban” OR “geographic” OR “inner city”, “disparity” OR “underserved” OR “equity” OR “vulnerable”, OR “poverty” OR “income” OR “social determinant” OR “resource-limited” OR “socioeconomic status” OR “minority” OR “racial” OR “cultural” OR “ethnic” to increase the scope of the review. All review articles were excluded from the results.

Data Items

The first and second authors extracted data based on the three domains of interest (race, SES, and geography). The data also include several related subcategories, such as ethnicity, socioeconomic factors (income, transportation availability, parental education, public assistance, parental employment, and education), insurance (private, public, or lack of insurance), family location (urban or rural, as defined by the U.S. Census), healthcare utilization (primary care, emergency department, telehealth, outpatient care, community care, and specialty centers), and barriers to care due to attitudes/beliefs relating to race or diagnosis (from the patient perspective or the provider perspective). Excel was used to keep track of the dataset.

Critical Appraisal of Individual Sources of Evidence 

A final critical appraisal of articles after the tier two review process was undertaken to ensure no bias was present in the final selection. The first and second authors divided the 19 articles that were selected by the tier two review authors and began the appraisal using the Joanna Briggs Institute (JBI) critical appraisal checklist for the analytical cross-sectional studies tool. Articles were included if they had a risk of bias that was minimal, with scores above 70% validated by both authors. Two articles were further excluded after following the checklist, one due to a lack of clarity on methods used for the population and the second due to a lack of detail provided for statistical analysis.

Synthesis of the Results

An initial data summary table was prepared by identifying details from each article demonstrating the correlations of any of the three domains to health-related outcomes or reported outcomes of the patient. Using these details, the articles were assigned to one of three groups: negative outcomes reported (i.e., low SES correlates with poor health-related outcomes) positive outcomes reported correlation (i.e., AA race correlates with better health-related outcomes), and no correlation (i.e., rural living is unrelated to health-related outcomes). In some cases, no correlation was made between any of the measures reported in an article, though at least one variable was tracked. In other cases, multiple aspects of a single overarching variable (i.e., SES) were tracked with differing correlations reported (i.e., some positive findings, some negative findings). Studies were included in relationship mapping only if a domain was correlated to health-related outcomes and not merely mentioned as a descriptor.

Results

PRISMA Flowchart and Data Extraction Process

**Figure 1:**
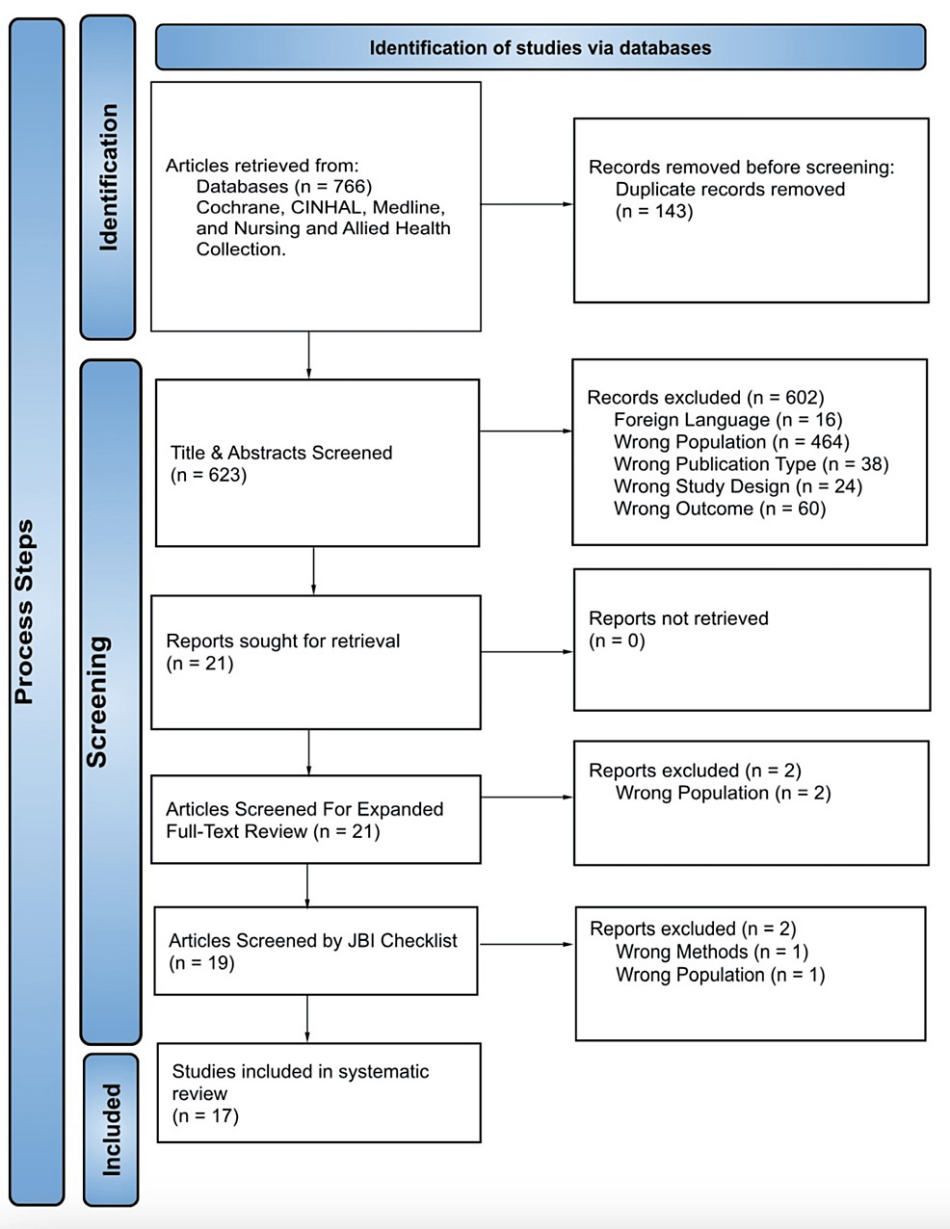
PRISMA flow diagram PRISMA flow diagram detailing the article screening process used in the current study; PRISMA: Preferred reporting items for systematic reviews and meta-analyses

**Figure 2:**
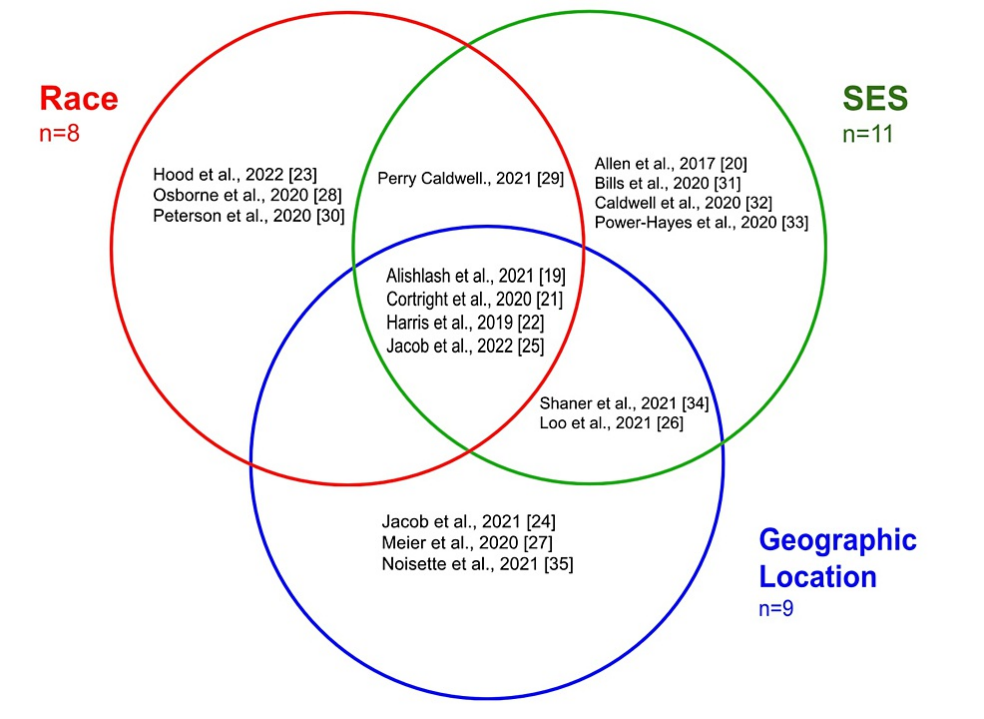
Venn diagram analysis by domain Relationship mapping of selected studies based on the domains of race, SES, and geography. Studies were included in relationship mapping only if a domain was correlated to health-related outcomes and not merely mentioned as a descriptor or demographic. Seven studies that were diagrammed covered more than one domain of analysis, exemplifying multifaceted barriers to care.

The initial search identified a total of 766 citations. Duplicates were removed (143 articles), which left a total of 623 unique studies to be screened. Seventeen articles met all the previously defined inclusion and exclusion criteria for inclusion in the study, as depicted in the PRISMA flowchart in Figure 1. Data were extracted from the 17 included articles into one of three domains - race, SES, and geography. Owing to the complex interactions between these variables, many studies examined more than one variable at a time. The Venn diagram in Figure 2 exhibits the categories evaluated by each study, and these categories are further broken down into subcategories within Table 1. It is of note that a study may have included a variable in their initial population summary/demographics, but not as a key element of analysis or conclusion. This is reflected in the data table, with a (*) denoting that the study included the domain in demographic terms, but not in the articles’ conclusions or data correlations. The articles denoted with an (*) were included to avoid bias in terms of inclusion/exclusion from criteria and to provide an accurate representation of population demographics. Of the 17 final selected articles for review, eight reported race as a key aspect of investigation and correlation, 11 reported SES, nine reported geographical location, and seven explored more than one domain. Table 2 lists the details from each article relating to the three domains of interest, as well as the assignment of each article as reporting a positive, negative, or neutral impact on health-related outcomes. Representing the data in this format helps to illustrate the interconnected nature of the variables and the overlap in them between studies.

**Table 1:** Data extraction Characteristics of selected studies evaluating parameters relating broadly to patient race, socioeconomic status (SES), and geographic residence. An asterisk (*) indicates study mentioned the domain but not in primary analysis/correlation/conclusion.

Measure type	Subcategory (if applicable)	Subcategory (if applicable)	Citations
Race/Ethnicity	Reported Race/Ethnicity		Alishlash et al., 2021 [19]
			Allen et al., 2017 [20]*
			Cortright et al., 2020 [21]
			Harris et al., 2019 [22]
			Hood et al., 2022 [23]
			Jacob et al., 2021 [24]*
			Jacob et al., 2022 [25]
			Loo et al., 2021 [26]*
			Meier et al., 2020 [27]*
			Osborne et al., 2020 [28]
			Perry Caldwell et al., 2021 [29]
			Peterson et al., 2020 [30]
	Bias/Discrimination/Inequality	Provider Perspectives	N/A
		Patient Perspectives	Alishlash et al., 2021 [19]
			Hood et al., 2022 [23]
			Osborne et al., 2020 [28]
		Parent Perspectives	Jacob et al., 2022 [25]
SES	Income		Alishlash et al., 2021 [19]
			Bills et al., 2020 [31]
			Caldwell et al., 2020 [32]
			Cortright et al., 2020 [21]
			Harris et al., 2019 [22]
			Loo et al., 2021 [26]
			Perry Caldwell et al., 2021 [29]
			Power-Hays et al., 2020 [33]
			Shaner et al., 2021 [34]
	Parent/Caregiver Education/Cognitive Development		Alishlash et al., 2021 [19]
			Bills et al., 2020 [31]
			Caldwell et al., 2020 [32]
			Hood et al., 2022 [23]*
	Patient Education/Cognitive		Allen et al., 2017 [20]
			Bills et al., 2020 [31]
			Harris et al., 2019 [22]
	Food Insecurity/Assistance		Loo et al., 2021 [26]
			Power-Hays et al., 2020 [33]
	Parental Employment		Jacob et al., 2022 [25]
	Transportation Barriers		Jacob et al., 2021 [24]*
			Jacob et al., 2022 [25]
			Loo et al., 2021 [26]
	Housing Instability		Alishlash et al., 2021 [19]
			Power-Hays et al., 2020 [33]
	Household Structure	Single Parent/Dual Parent/Non-Traditional	Cortright et al., 2020 [21]
	K-12 Education		Jacob et al., 2022 [25]
	Public/Private Insurance		Alishlash et al., 2021 [19]
			Harris et al., 2019 [22]
			Meier et al., 2020 [27]*
			Power-Hays et al., 2020 [33]
Geographic Location	Reported Rural or Urban		Alishlash et al., 2021 [19]
			Cortright et al., 2020 [21]
			Harris et al., 2019 [22]
			Jacob et al., 2021 [24]
			Jacob et al., 2022 [25]
			Loo et al., 2021 [26]
			Meier et al., 2020 [27]
			Noisette et al., 2021 [35]
			Shaner et al., 2021 [34]
	Health Utilization Modality	Emergency Department (ED)	Cortright et al., 2020 [21]
			Peterson et al., 2020 [30]*
			Shaner et al., 2021 [34]
		Outpatient	Loo et al., 2021 [26]
			Power-Hays et al., 2020 [33]*
			Shaner et al., 2021 [34]
		Telehealth	Jacob et al., 2022 [25]
			Noisette et al., 2021 [35]
			Shaner et al., 2021 [34]
		Main Hospital (Inpatient)	Shaner et al., 2021 [34]
		Specialty Clinic	Loo et al., 2021 [26]
			Noisette et al., 2021 [35]
		Satellite Clinic	Shaner et al., 2021 [34]
		Telemedicine	Jacob et al., 2021 [24]
			Jacob et al., 2022 [25]

**Table 2:** Summary table Seventeen studies were included in the scoping review and analyzed for data relating to the three domains of race, SES, and geographic location. The general findings of each study were summarized, as well as the associated limitations of each study. Articles that correlated one or more domains with health-related outcomes were identified for further grouping of findings of positive, negative, or neutral outcomes. Two studies evaluated populations that were slightly older than 18 years, but were included as the studies did still incorporate the target population and expanded the natural history of the population for educational attainment measures through high school. SES - socioeconomic status; SCD - sickle cell disease; ACS - acute coronary syndrome; ADHD - attention-deficit/hyperactivity disorder; ED - emergency department; SDOH - social determinants of health; AA - African American; NIS - national inpatient sample; NEDS - nationwide emergency department sample; ICD-9 - International Classification of Diseases, Ninth Revision; HCUP - Healthcare Cost and Utilization Project; HMH - household material hardship; US - United States

Citations	Study Design	(n=#)	Child age (years, months reported when applicable)	Setting	Study aims	Healthcare delivery factors affecting SCD care	Findings	Limitations
Alishlash et al., 2021 [19]	Retrospective cross-sectional study	n=709	Mean age:13.6, Range: not reported	Hospital - Children's of Alabama	Evaluate the correlation between pediatric SCD patients experiencing ACS and neighborhood socioeconomic deprivation and racial composition.	Race, SES, geographic location	Incidence and recurrence of ACS in the pediatric SCD population were significantly lower in communities that were socioeconomically deprived and those that were of predominant AA makeup, indicating protective effects and comfort of ethnic density in close-knit communities. There was no correlation between rural living and ACS.	This study suffers from four main limitations, including the cross-sectional study design which prevents a determination of causality, potential information bias with admissions data, inability to control for environmental factors that may impact pulmonary function, and lack of data regarding individual socioeconomic factors.
Allen et al., 2017 [20]	Cross-sectional observational study	n=45	Mean age: 12.3, Range: 8-16 yrs	Outpatient - Pediatric Sickle Cell Clinic in the Southeastern US	Evaluate the association between quality of life and cognitive measures like executive functioning in SCD patients as well as the psychosocial and caregiver factors involved.	SES	Executive functioning and cognitive ability had a strong correlation with self-reported quality of life; however, SES did not play a role in health-related outcomes for either patients or parents.	Two main limitations to this study include a lack of objective neurocognitive measures and a small sample size of 45.
Bills et al., 2020 [31]	Cross-sectional observational study	n=70	Mean age: 6.47, Range: 4-8 yrs	Outpatient - Pediatric hematology/oncology clinic in the Southeastern US	Evaluate cognitive and behavioral measures in pediatric SCD patients with a potential relationship between socio-environmental factors.	SES	Low SES had a negative impact on reading, mathematic proficiency, social ability, language skills, and mental impairment such as ADHD. SES and parental/family functioning were identified as the primary factors negatively impacting these health-related outcome measures.	Three key limitations to this study include potential information bias with parents self-reporting data, a limited scope of environmental risk factors measured, and the inability to control for variables with certain factors measured.
Caldwell et al., 2020 [32]	Cross-sectional, descriptive, correlational study	n=59	Mean age: 14.91, Range: 10-18 yrs	Outpatient - Tertiary care center in Dallas, TX	Evaluate the relationship between health literacy levels in pediatric SCD patients and factors such as caregiver health literacy and socioeconomic traits.	SES	This study found no significant correlation between caregiver healthcare literacy and adolescent SCD patient health literacy, nor was there a significant relationship between healthcare literacy and SES factors when other factors such as age were controlled for.	Limitations to this study include a relatively small sample size of 59 patients and caregivers, sample bias based on when patients were recruited, and limited scope of measures evaluated as the study was an initial approach.
Cortright et al., 2020 [21]	Retrospective cross-sectional study	n=126 (52 Visited the ED in the last 12 months and 74 who did not). The average age in the ED group was 6.15 with a range of 4-7 while the average age in the non-ED group was 8.56 with a range of 7-10	Mean age ED Group: 6.15, Mean age non-ED Group: 8.56, Range: ED Group: 4-7, Range: Non-ED Group 7-10 yrs	Hospital - EDs across the US	Two aims of this study include the relationship between SDoH and ED visits in pediatric SCD patients. Secondarily, this study evaluates the association of these SDoHs with pediatric SCD hospital admission from the ED.	Race, SES, geographic location	This study revealed that the only SDoH associated with ED visits in pediatric SCD patients is household structure (i.e. married parents, single mother, single father, etc.). There was no association between commonly studied factors such as SES, racial identity and region of residence which the article attributed to an inherent disadvantage within the community that dissuades ED visits and admissions.	Several limitations can be seen in this study with a lack of data regarding SCD genotypes, additional confounding mental health comorbidities, additional emergent medical settings (i.e. urgent care), and lack of control of SDoH such as access to transportation.
Harris et al., 2019 [22]	Retrospective cohort study	n=108	Mean age: 20.2, Range: 16-25 yrs	Hospital - St. Louis Children's Hospital	The purpose of this study includes the evaluation of rates of education in pediatric patients with SCD, environmental factors affecting educational attainment in these patients, and the prospective potential for an educational program to aid pediatric SCD patients.	Race, SES, geographic location	Overall, patients with SCD or SCD stroke history graduated at higher rates than their AA peers. Patients living in areas with greater patient density achieved higher levels of education than patients living in lower-density areas. SES was not related to patient educational achievement.	The design of this study is its major limitation, as it is a single-center retrospective study, resulting in a lack of generalizability. Additionally, there were no measures regarding potential mental health comorbidities affecting these patients.
Hood et al., 2022 [23]	Mixed methods- observational & retrospective	n=30	Mean age: 11.3, Range: 8-16 yrs	SCD clinic at Children's Hospital of Alabama	Measure the relationship between health-related stigma and racial bias on health-related outcomes measures	Race	Younger age is significantly related to increased perceived racial bias. Increased health-related stigma and perceived racial bias were related to poorer health-related outcome measures. High levels of perceived racial bias had a particularly strong negative influence on older black females with SCD.	Small sample size. Single institution. Self-reported methods instead of interviews, would have allowed for more exploration of participants' emotions related to stigma and racial bias. Caregivers were present during the survey, possibly leading to influence. Many patients were currently receiving chronic transfusion therapy and reported poor-to-moderate health-related outcomes measures.
Jacob et al., 2021 [24]	Cross-sectional, observational, feasibility study	n=10	Mean age: 8.5, Range: 10 months - 18 yrs	Pediatric Sickle Cell Clinic at Riley Hospital for Children in Indianapolis, Indiana	Evaluate the feasibility of utilizing telemedicine as a healthcare delivery tool for pediatric patients with SCD in a rural and medically underserved area.	Geographic location	Telemedicine was initiated as a mechanism to improve follow-up with patients with SCD living in rural Indiana, thereby neutralizing the negative impact of distance to specialized care on health-related outcomes measures. 60% of participants had been lost to follow-up or did not attend >50% of scheduled sickle cell visits prior to beginning telemedicine visits. Following telemedicine implementation, 9/10 participants participated in telemedicine visits with 100% follow-up. Patient satisfaction was reported as good or excellent for all participants.	A Small sample size. Single rural medically underserved areas, with telehealth services that might differ in other rural or urban areas. Patient satisfaction scores were not compared to patients who used in-person clinics.
Jacob et al., 2022 [25]	Observational-qualitative interviews	n= 16 Caregivers of children with SCD	Mean age: Not reported, Range: Not reported	Pediatric Sickle Cell Clinic at Riley Hospital for Children in Indianapolis, Indiana	Understand the facilitators and barriers to care and the perception of telemedicine by caregivers of pediatric SCD patients in a medically underserved area.	Race, SES, geographic location	Interviews revealed racial biases and the negative impact of financial strain on health-related outcome measures. Telemedicine and satellite clinics were able to neutralize some of the impact of distance from specialty care and most parents had positive experiences. However, challenges with rescheduling and the lack of physical exams remain barriers to good health-related outcomes for patients living in rural areas.	Small and focused sample size. Study included caregiver interviews and did not assess patient feelings. Study did not include further differences in SES status which could further detail barriers to care among this population.
Loo et al., 2021 [26]	Observational-qualitative interviews using focus groups	n=46 Pediatric hematology clinic staff	Mean age: Not reported, Range: Not reported	6 focus groups (4-10 participants) at four urban pediatric hematology clinics in the Northeast region of the U.S.	Assessing pediatric hematology staff and providers' perspectives regarding barriers and facilitators in addressing unmet basic needs for children with SCD	SES, geographic location	Medical staff reported the large socioeconomic burden on their patient population that was unique to those with SCD compared to other patient populations in the clinics. Urban residents with access to specialty clinics still suffered from the long distances to specialty pharmacies for hydroxyurea formulations.	Sample size limited to 4 SCD clinics. Focus groups used a cost- and time-effective way to collect information but could have led to group-think.
Meier et al., 2020 [27]	Retrospective chart review	n=198 Children with SCD in Indiana participating in Sickle SAFE for >1 year	Mean age: Not reported, Range: 2 days - 3 years	Sickle SAFE Program of Indiana, a voluntary 3-year program for infants born with SCD providing education and medical guidance using in-home visits and telephone follow-up.	Assessing the proportion of children who received transcranial Doppler (TCD) screening, influenza, and pneumococcal vaccination, were prescribed hydroxyurea, genetic counseling, and time to receipt of penicillin prophylaxis.	Geographic location	Patients participating in the Sickle SAFE Program received penicillin prophylaxis before 2 months of age regardless of urban/rural living. Rural counties had lower rates of Pneumococcal polysaccharide vaccine, transcranial Doppler ultrasound screening and hydroxyurea prescription compared to urban counties.	The voluntary nature of the Sickle SAFE program introduces potential selection bias, impacting the generalizability of findings. Regional factors - with the selection of participants limited to Indiana - also lead to issues of comparison to other regions.
Noisette et al., 2021 [35]	Observational	n=24 Providers from 28 SCD centers	Mean age: Not reported, Range: Not reported	DISPLACE (Dissemination and Implementation of Stroke Prevention Looking at the Care Environment), an NHLBI-funded study including 28 sites across the U.S. looking to improve monitoring care in SCD.	Impact of COVID-19 on care focusing on Transcranial Doppler ultrasound (TCD), chronic red cell transfusions (CRCT), telehealth, and COVID-19 testing.	Geographic location	Reports of services for patients during the height of the COVID-19 pandemic indicated the broad implementation of telemedicine to minimize interruptions of care for patients with SCD. However, TCD rates declined dramatically during the reporting period particularly with sites in the East and Midwest, as well as smaller sites, less likely to offer SCD screens.	Survey of only DISPLACE participating sites, and not all university clinics treating SCD. Surveys reflected adjustments made in medical institutions and may not measure the impact of the pandemic on caregivers and patients. Results are descriptive and do not provide efficacy of changes regarding COVID.
Osborne et al., 2020 [28]	Descriptive cross-sectional study	Aim 1: n=5 Children-parent dyads from HABIT and n=5 Children-parent dyads from Columbia University Medical Center Aim 2: n=28 dyads in HABIT study	AIM1 children's mean age: 10.8 ± 1.1 and 16.0 ± 1.0 yrs. Parents' mean age: 35.2 ± 7 and 38.6 ± 8.1. AIM2 children's mean age: 13.6 ± 2.4 yrs. Parents' mean age: 42.9 ± 9.3 yrs	New York Presbyterian/Columbia University Medical Center Transplant Center and Columbia’s Pediatric SCD Clinic.	Aim 1: Evaluate an English-Spanish translator for children with SCD aged 8-12 and 13-18 years old and their parents. Aim 2: in Latino and non-Latino populations, compare generic vs disease-specific quality of life factors and influences.	Race	Families of and patients with SCD who identified as either Latino or non-Latino AA were compared for health-related outcomes. While Latino parents were more worried about their child with SCD having a stroke than non-Latino AA parents, they displayed less anger regarding the diagnosis, were more satisfied with their child’s medical treatment and were able to communicate better about their child’s disease compared to non-Latino AA patients/families.	The study may not be generalizable because it was based out of two locations in New York, the study is not representative of all factors influencing the quality of life, and there are limited studies conducted on the quality of life between chronic disease patients of Latino and non-Latino origin.
Perry Caldwell et al., 2021 [29]	Cross-sectional, descriptive, exploratory study	n = 239 (134 with SCD and 105 without SCD)	Mean age with SCD: 14.8 ± 2.17 yrs, Mean age without SCD: 15.87 ± 2.33 yrs, Range: 10-19 yrs	Patients recruited at a tertiary center in a large metropolitan area for patients with SCD. Patients recruited across the state of Texas online for patients without SCD.	Compare factors influencing and differences between patients with SCD and those without in relation to health literacy	Race, SES	There was a positive correlation between health literacy and age/grade level for patients with SCD, but there was no correlation between health literacy and parental income and education level. Black patients with SCD scored similarly on measures of health literacy to their Black peers without SCD. However, Blacks scored lower than Whites on health literacy in the control group, thus the patient group scored lower than the control group, indicating an impact of race independent of diagnosis.	Convenience sampling was used. Different recruitment tools were used for patients with SCD vs patients without SCD. This could be a potential new variable between eHealth literacy and health literacy.
Peterson et al., 2020 [30]	Serial cross-sectional, retrospective population-based study	n = 70,033 Encounters on average per year from January 1, 2006, to September 30th, 2015 who had conditions related to sickle cell	Mean age: Not reported, Range: Not reported	NIS records 7 million national hospital visits and NEDS records 31 million national ED visits	Trends involving patients with SCD and Sickle Cell Trait (SCT) in acute in-patient and emergency settings.	Race	75% of those in the hospital with SCD were non-Hispanic (NH) Blacks. HbSS was the most common subtype found in hospitalized SCD patients. Patients with HbSS were NH-White 1.0%, NH-Black 79.5%, Hispanic 4.4%. Patients with SCT were NH-White 6.1%, NH-Black 66.3%, Hispanic 7.4%.	HCUP accounts for encounters, not for a patient over their life. Inappropriate use of ICD-9 codes relating to SCD.
Power-Hays et al., 2020 [33]	Retrospective EMR review	n = 115 (101 eligible)	Mean age: 8.7 yrs, Range: 12 months - < 18 yrs	Boston Medical Center pediatric hematology clinic using SDoH screener	The effects of HMH on ED usage by patients with SCD.	SES	59.4% of families of patients with SCD reported at least one HMH. Having one or more HMH was correlated with higher ED reliance.	Only patients who completed an SDoH screener at least one time were included, data had to be collected the year prior to a patient filling out the SDoH because once an HMH was marked patients were given resources to help them, data is limited to a representation of the patient population at Boston Medical Center.
Shaner et al., 2021 [34]	One-year retrospective review	n = 172 HbSS and HbSbO thalassemia	Mean age: 11 ± 5 yrs, Range: 6-16 yrs	Receiving care at The University of Alabama Birmingham academic center or satellite campuses	Patients using telehealth in rural areas for hydroxyurea dosage adjustment at satellite campuses vs university-based patients.	SES and geographic location	There was no relationship between SES or clinic location and hydroxyurea utilization, though an increased number of clinic visits did correlate with increased hydroxyurea utilization. Satellite centers and telehealth can assist rural patients with SCA in receiving exceptional care; however, there are systemic barriers that affect satellite campuses such as not having as much freedom to reschedule appointments.	Recall bias to satellite campuses of acute visits. Could not confirm if patients were continued on a certain hydroxyurea dose, due to adherence after the visit. Individualized SES was not obtained, data was derived from the patient's zip code.

Race

**Figure 3:**
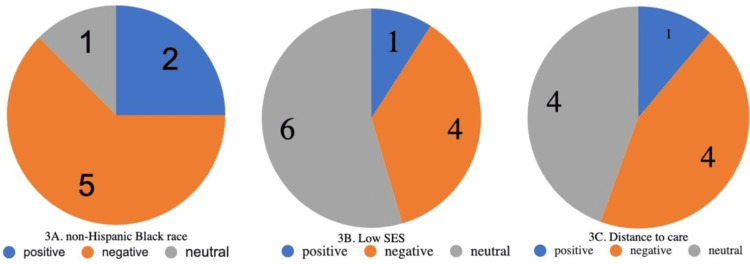
Correlations between domains and health-related outcomes Outcome measures from each study were evaluated for reporting a positive impact of the variable (blue), a negative impact of the variable (orange), or a neutral/no impact of the variable (gray) on health-related outcomes. A) Indicates the proportion of articles that studied patients of non-Hispanic Black race, health-related outcomes, and the reported impact. B) Indicates the proportion of articles that studied patients of low socioeconomic status (SES), health-related outcomes, and the reported impact. C) Indicates the proportion of articles that studied patients living long distances (in either rural or urban settings) from specialized care, health-related outcomes, and the reported impact.

Many of the studies included race as a statistic, as well as racial discordance between healthcare providers and patients. Eight studies (8/17, 47%) specifically analyzed race as a factor in health-related outcomes and experience. Figure 3A groups the articles based on reports of correlations between racial identity as non-Hispanic Black and health-related outcomes. Health-related outcomes were defined as a term that encompassed holistic barriers to care accessibility, attainment, and delivery in the SCD population within the context of race, SES, and geographical evaluation. Five of these studies reported negative health outcomes associated with race, while one reported neutral association of race and two others reported positive associations.

Five studies reported racial identity correlating with poor health-related outcomes, including race/racial bias [23,25,28-30]. It is of note that younger age in this pediatric population was significantly related to increased perceived racial bias [23,24]. An evaluation of acute care utilization nationwide reported that 75% of children hospitalized with SCD were non-Hispanic Blacks (NHB), and 1% were non-Hispanic Whites (NHW), yet patients seeking emergency medical care for sickle cell trait-related illnesses were 66% NHB vs 6% NHW - with inpatient admissions through the ED accounting for the largest medical expenditure of the SCD subtypes [30]. In an acute care setting, AAs were prescribed lower doses of pain medication (if prescribed at all) compared to their white counterparts while reporting a higher chronic pain score [23]. Caregivers of children with SCD also reported concern about provider racial bias [25]. In interviews, a loss of trust in providers was reported due to a lack of knowledge of SCD-specific care and blatant bias that impacted the care of patients [25]. Unique among the reports was a comparison of the health-related outcomes of patients with SCD who were Latino versus those who were non-Latino, with higher health-related outcomes reported for Latino parents and patients [28]. Another report examined the concept of health literacy, which was validated via the “Newest Vital Sign health literacy instrument.” The term health literacy is used to estimate a patient’s ability to use information and navigate services and resources to inform their healthcare decisions. This illustrated that Black, non-Hispanic children with SCD had decreased health literacy compared to White children without SCD but similar health literacy as Black children without SCD [29]. In a discussion of healthcare utilization and outcomes, one study reported that increased ED utilization was not correlated with race for patients with SCD, thus suggesting a neutral association regarding health-related outcomes [21]. It is of note, however, that this study used a population-based design, and as a trend, AA children were previously reported to utilize EDs for care, which often provide less comprehensive and continuous care when compared to specialty clinics.

One additional study reported a neutral association with race finding that AA children with fair or poor caregiver-related health were more likely to be admitted following an ED visit [21]. However, these results did not reach statistical significance and instead pointed to household structure as a predictor of outcome. Two other studies reported positive health-related outcomes relating to racial identity as AA. Researchers found a lower incidence and recurrence of acute chest syndrome (a serious complication of SCD) in children who lived in areas of high ethnic density, and separately found patients with SCD living in ethnically dense areas achieved higher levels of education [19,22].

SES

The most frequent domain reported was SES (11/17, 65%). Figure 3B groups the articles based on reports of correlations between SES- and health-related outcomes. Eleven studies focused on the impact of SES on care and outcomes in the pediatric population of SCD patients [19-22,25,26,29,31-34] - four showed negative associations with low SES, six showed neutral relation to low SES, and one showed a positive relationship with low SES. Among those that discussed SES along with other factors, the most common coinciding topics include race, location, and familial barriers, as discussed below.

Four studies reported a correlation between measures of low SES status and poor health-related outcomes [25,26,31,33]. Within the SCD population, it was suggested by Bills et al. that SES may predict language-related outcomes, specifically semantic, syntactic, and early reading skills in this population [31]. These results were further correlated to measures of “parent and family functioning” (parental depression, home involvement/parental support of academic and socialization/cognitive skills, parental engagement, positive parent-child interactions, and two parent-report measures to assess parent and family distress), which influenced cognitive and behavioral outcomes not predicted by SES [31]. This illustrates the distinct and independent influence of SES. SES was observed through a different lens as “household material hardships,” including factors such as poverty, food insecurity, and unstable housing [33]. The article concluded that decreased SES has an association with an increased percentage of ED reliance, which is widely shown to lack of specialty care required by this population [33]. From a provider perspective, SES was examined alongside multifactorial barriers to care in patients with SCD. Many providers reported frustration regarding the “unequal distribution of resources” across different subspecialty clinics, especially those serving the SCD population [26]. Providers felt restricted in the amount of assistance they could offer patients, as there were so many different factors involved in why a patient may not be able to access the care they need [26]. Families also noted the double burden of financial strain and missed days at work as variables that impacted access to care [25].

Six of the studies found a neutral correlation between measures of low SES and poor health-related outcomes [20-22,29,32,34]. The idea of “parent functioning,” as discussed above, was further described by Cortright et al. who found that within a national sample, a child with more ED visits was more likely to come from a single-family home [21]; however, there was a lack of correlation between SES and ED utilization. Lower SES did not directly have an increased association with ED usage [21]. It is of interest that this article determined the inherent disadvantage of low SES to dissuade overall ED visits and admissions, which contrasts with earlier literature and other studies that associate poverty and lack of access to preventive care with increased ED reliance and worse outcomes [21].

Education (type and duration) was also included within the lens of SES. This is especially important in this population, as children with SCD are often at risk for future intellectual disabilities and memory impairment [36]. Four studies looked at SES factors, such as health literacy and education among parents and children, and the impact on their children’s health outcomes and overall quality of life [20,22,29,32]. Allen et al. reported that impairments in the executive and psychosocial development of the child were associated with lower health-related outcomes scores [20]. This study examined the domains of physical health, psychological health, and environmental factors (financial resources, health accessibility, physical environment, and social relationships/support). These additional variables had the potential to impact downstream employment, education, and socialization for pediatric patients with SCD [20]. Interestingly, while previous studies have demonstrated emotional well-being and caregiver SES as predictors of executive skill and attainment, Allen et al. reported psychosocial resources, including those of the caregivers, were not found to moderate the relationship between cognitive functioning and quality of life for patients with SCD [20]. In fact, Caldwell et al. demonstrated that, although caregiver income and highest education level did positively correlate with adolescents’ health literacy scores, the overall relationships were nonsignificant while controlling for each other, adolescent age, and the other variables investigated [32]. When compared to age-matched peers, Perry Caldwell et al. later found low literacy levels in patients with SCD compared to age and grade-level matched peers; however, only a relationship between annual household income and literacy was delineated in age-matched peers, not the sickle cell group [29]. Of note, although this study examined patients aged 0-19, it was included because it incorporated the target age range and provided a beneficial longitudinal comparison between groups of individuals [32]. A separate study showed no correlation between low SES and poor educational achievements for patients with SCD [23]. In addition, low SES was correlated to rural living but did not lead to poorer health-related outcomes [34].

Only one study reported a positive association between lower SES and quality of life [19]. Acute chest syndrome, a complication of SCD, was lower in ethnic, close-knit AA neighborhoods, even in the setting of lower SES and higher family stress [19]. This was credited to what the article deemed the protective “ethnic density effect” pointing to the importance of neighborhood demographics [19]. This was a significant finding, as most of the literature up to this point had found that acute complications of SCD are the highest in areas with lower SES; however, generalization is difficult given this study's location within rural and urban areas of Alabama specifically. Notably, Shaner et al., as discussed above, indicated that, though rural patients had lower SES and more difficulty accessing/attending/and rescheduling appointments, leading to systematic barriers to hydroxyurea treatment compliance and responsiveness, there was still no correlation between low SES or rural living and health-related outcomes [34].

Geographical Location

Nine studies (9/17, 53%) discussed the impact of the geographical location of the patient’s residence on healthcare delivery and outcomes in the pediatric population with SCD. Figure 3C groups the articles based on reports of correlations between distance to specialty care and health-related outcomes. One study reported positive associations with distance to care, four reported negative impacts, and four reported neutral impacts. Increased distance to travel to specialized care was correlated to poor health-related outcomes in four studies [25-27,35]. These studies found that proximity to care meant proximity to trained staff and resources and distance from specialty compounding pharmacies limited hydroxyurea utilization [26]. Patients living near specialty clinics located in urban centers had improved vaccination rates, stroke screening, and hydroxyurea utilization compared to patients living in more rural locations [27]. Satellite centers and telehealth monitoring (especially during the height of the COVID-19 pandemic) brought care to rural communities, but patients did not have as much freedom to reschedule appointments or were unable to receive physical exams, with regional differences in the US noted [25,35].

Four studies indicated rural living was not correlated with poor health-related outcomes. While rural living was correlated to lower SES, there was no correlation to poor health-related outcomes simply based on rural location [19,34]. The articles did report a positive correlation between the number of clinical visits and health-related outcomes, which could ultimately impact patients at a greater distance to clinics [34]. There were no regional differences in ED utilization [21]. Telehealth and satellite clinics were reported to play a potential role in neutralizing the negative effects of distance on health-related outcomes [19,21,24,25]. Rural patients utilizing telemedicine showed increased follow-ups after initial evaluation at a specialty center [28]. Importantly, geography impacted health-related outcomes in a way not defined by distance, but rather by ethnic density and diagnostic density of the area. Similarly, there was a lower incidence and recurrence of acute chest syndrome in children who lived in areas of high ethnic density [19].

While geographic location impacts patients’ distance to care, it can also impact other multidimensional factors that may influence overall outcomes. Harris et al. [22] found patients living in high patient concentration areas were found to have accomplished higher educational attainment, likely due to being in urban locations, which have more access to education. These high patient concentration areas were likely to be in an urban environment and to have a higher percentage of residents identifying as AA (p<0.05) [27]. The aspects of race, education attainment, and health literacy are reported in previous sections. However, this is important to note here as geographical location can impact proximity to education, resource access, skilled care, and those with a shared racial identity, which all result in a patient’s overall outcome.

Discussion

This study sought to understand how racial identity, SES, and geography affect access to healthcare and outcomes in children with SCD in the US. The 21st century has seen a rapidly changing healthcare landscape with variables such as the COVID-19 pandemic, increased access to health insurance through the Affordable Care Act (ACA), the increasing popularity of telehealth platforms, and social movements such as “Black Lives Matter.” SCD predominantly impacts AAs, and a current evaluation of these factors is necessary. To that end, this scoping review examined articles from 2017 to 2022, a crucial five-year period of dynamic social change.

SCD primarily impacts the AA population in the US. Consequently, racial and social stigma may become intertwined with health-related stigma, which is defined as the "devaluation, judgment, or social disqualification of individuals or populations who identify with particular health-related conditions" [23,25]. Five studies provided evidence of an association between racial identity and poor quality of healthcare administered, potentially leading to a cyclic relationship founded on mistrust between AAs and healthcare workers, especially in majority-white healthcare systems [23,25,28-30]. There are reports of stigmatizing language such as "frequent flier," “clock watcher,” “drug seeker,” and “sicklers,” which perpetuated the divide between patients and healthcare workers [23,25,29]. Further research is needed to assess how the stigmas specific to patients with SCD impact healthcare outcomes, as well as how exposure to this stigma at a young age impacts disease outcomes, especially given the racial predominance seen with this disease. Perry Caldwell et al. reported that race may play an unclear but significant role in adolescent health literacy, illustrating another health-related disparity [29]. It is unclear how the variable of race in isolation itself impacts ED utilization, and more research is needed.

When looking at race in patients with SCD, it can rarely have a positive impact on the outcome; however, patients living in areas of high racial/ethnic density may benefit from enhanced social support, a stronger sense of community, and neighborhood cohesion, which can show to be protective in outcomes [19,22]. This demonstrates the power of shared racial community, ethnic density, and support. Patients with SCD may have fewer socialization opportunities due to frequent hospital and/or doctor visits and may experience isolation within their community due to the inability to participate in certain activities. Enhanced social support could decrease reports of depressive symptoms, which were found to be higher in patients with SCD, especially those experiencing health-related stigma [19,22]. It is unclear how this concept may transfer to different settings of either rural or urban environments, as interpretation is limited by the study locations, which are specific to Alabama and St. Louis, Missouri. Further research is needed regarding the generalization of these findings to the broader US. Although racial bias is present within the healthcare system, these studies suggest racial belonging may also provide comfort when institutions do not. Further research is needed to assess how community composition affects the ability of a child with SCD to handle the emotional and physical toll of the disease.

The largest percentage of articles included in this study assessed the domain of SES, and it was more common to see a neutral/negative association between low SES and poor patient outcomes. According to the American Psychological Association, “Socioeconomic status is the position of an individual or group on the socioeconomic scale, which is determined by a combination of social and economic factors such as income, amount and kind of education, type, and prestige of occupation, place of residence, and-in some societies or parts of society-ethnic origin or religious background" [37]. In an alternative definition, seen in Baker et al., SES was described as “a measure of one's combined economic and social status and tends to be positively associated with better health" [38]. With these definitions and variables of inclusion, low SES was predicted to correlate with poor healthcare outcomes in alignment with earlier reports. However, this was not universally reflected in the current literature on this topic. In fact, the majority of articles reported a neutral effect of socioeconomic status in relation to outcomes, with some pointing to variables such as family structure and education as contributory/explanatory. This neutral effect may also be attributed to the rise of telehealth visits as a means to close the gap in care accessibility. These studies assume that those geographically located in a rural setting have less access to appointments and skilled care. Even though telehealth visits allow for increased contact and follow-up, there is something to be said about the quality of these visits since in-person physical examinations are not possible.

Of the studies that focused on SES in relation to health outcomes, many chose to evaluate household structure and its effects on healthcare utilization, as well as cognitive, behavioral, and/or medical outcomes of pediatric patients. Of the four studies that correlated low SES to poor outcomes, cognitive measures were discussed alongside familial functioning and SES with each exerting a unique sphere of influence [31]. This pointed to the inherent negative impact of low SES (regardless of familial functioning) on the development of a child, especially with SCD. Low SES was shown to increase ED reliance [33]. Unequal distribution and allocation of resources were seen to impact the care of SCD patients from the viewpoint of providers [26]. Additionally, families struggle to receive care and suffer from food insecurity, unstable housing, and unreliable transportation [25].

Studies that determined a neutral effect of SES on outcomes pointed to alternative factors such as home environment. It was found that, contrasting with what was reported above by Power-Hays et al. [33], there was a lack of correlation between SES and ED reliance, instead finding a correlation between SES and “parent functioning” [21]. This further illuminates the lack of consensus on variables impacting quality of life, especially in relation to emergency utilization by this population. Impairments in the psychosocial and executive development of a child were not shown to be correlated with the psychosocial resources of the caregiver [20]. This is further supported by Caldwell et al., who concluded that the overall relationship is non-significant between caregiver income and health literacy [32]. Ethnic density and patient density were seen as protective factors against the negative impact of lower SES [19].

SES contributes to, but does not fully capture, the risks of negative psychological outcomes in children with SCD. The variable of SES is highly contested in its isolated importance in outcomes in this pediatric population and includes the multifaceted, direct, and indirect interplay of a plethora of variables. Some of the data suggests that areas such as parental involvement and mental health can be more predictive of health-related outcomes than SES and poverty, whereas other studies suggest that material hardships have correlations with a lack of specialty care and ED reliance. The literature reveals a complicated and multifactorial relationship between SES and healthcare outcomes for children with SCD. This was unanticipated, as previous research predicted a more linear relationship as reflected in a vast amount of earlier literature on the topic. Some of the discrepancies may be related to changes in society such as the ACA over the past decade and are now reflected in the current literature.

The geographical location of residence was another variable examined as a potential barrier to healthcare in children with SCD. We hypothesized that living in a rural location (increased distance to specialized care) would be correlated with poorer health outcomes due to less access to care. Surprisingly, there were no reported regional differences in ED utilization [21]. Of the nine studies that discussed geography, four of these reflected the importance of telemedicine in providing access to healthcare for patients with SCD and their families. Telehealth is of particular importance following changing patterns of care delivery in part due to the COVID-19 pandemic. Within a virtual environment, it would be expected that care could potentially be equivalent regardless of location in the country. However, despite the advent of telehealth, there were regional differences and site location capabilities for screening and virtual services offered [35]. In a broader discussion, two studies correlated the use of telemedicine visits with higher retention rates of patients with SCD, even in patients who were previously inconsistent in attendance or lost to follow-up appointments [24,35]. However, within two separate studies, Jacob et al. reported that many caregivers reported distance as a barrier to care and felt that telemedicine was not an adequate solution due to technical difficulties and the lack of physical examination [24,25].

Shaner et al. recognized that, although both telehealth and the existence of satellite campuses in more rural areas can assist patients with SCD in receiving care at a distance, there were systematic barriers associated with satellite centers, such as a lack of freedom to reschedule appointments when needed [34]. The administration of hydroxyurea varied between different counties, with patients in rural zip codes receiving fewer prescriptions than in other locations [27]. Provider commentary on unmet basic needs within this population highlighted that hydroxyurea is often difficult to access based on specific location [26]. Additionally, patients enrolled in the Sickle SAFE Program residing in the local urban area had improved vaccination rates and stroke screening compared to those in rural counties [27].

Variation in care outcomes based on geography was investigated through an alternate lens, as Harris et al. [22] correlated counties with higher populations of AA patients and SCD patients’ diagnoses to higher graduation rates from high school. This is likely due to the support received from patients and families in similar health situations [22]. There was also a lower incidence and recurrence of acute chest syndrome in children who lived in areas of high ethnic density [19]. From these particular studies, it appears that, while geography does play a role in access to care, other factors such as relationships and community support must also be considered in the outcomes of pediatric patients with SCD. Telehealth utilization has grown exponentially since the COVID-19 pandemic and therefore research in this area may not be fully captured in the dataset of this review.

Limitations

The primary limitations of this review are the sparse number of appropriate studies identified and the small sample size evaluated in many of the reports. In seven studies, the sample size ranged from five to 70 participants, including predominantly one race, or participants from one area of the US. Studies that only included participant data from associated sickle cell clinics might have sampling bias. Many of the studies did not specifically define the SES of the population they were sampling from and the definition of “rural” was subjective to the researcher conducting the investigation.

It was difficult to isolate single variables in each study, creating the potential for confounding variables within the literature itself. With the interconnectedness of factors that contribute to SES (income, education, occupation, place of residence, race, ethnic origin, religious background, etc.), it is difficult to categorize studies based on one topic of interest alone, as these variables do not occur in isolation. Racial identity and geography pose similar obstacles as research often lacks consensus on what is meant by words such as “Black”, “rural”, or “urban.” Similarly, in relation to race, stigma and stereotyping have the power to permeate a multitude of health outcomes, and their lack of reporting does not mean that there is no influence. Although a study may have collected data on multiple domains, their results and discussion often made no reference to them, further confounding proper data analysis. Consequently, a significant limitation of this study is the reliance on what was reported by the authors of the cited studies, making it difficult to compare results across studies and create a holistic view of a multifaceted disease model.

The article selection process used a single search via EBSCO hosts, including Cochrane, CINHAIL, Nursing and Allied Health Collection, and Medline databases, potentially excluding work reported in specialty journals. An expanded database search might have offset this limitation. Only articles published within the last five years were included, but these reported data collected from even earlier dates. Thus, these criteria actually capture not only current practice but also some of the early stages of the implementation of the ACA and the growing pains of telehealth operations. Overall, the limited database search resulted in a restricted assessment of factors that affect healthcare for pediatric patients with SCD.

Implications for Practice

Higher SES does not consistently equate to better healthcare outcomes, and improvements in SES may not correlate to improved health-related outcomes for pediatric patients with SCD. There were significant effects of parental factors, such as stress, depression, and a feeling of belonging. These variables have the potential to significantly impact the quality of care received by patients. Resources for parental stress management and low-cost counseling to combat depression and burnout that can come following a diagnosis of SCD could prove worthwhile for this population. Supportive groups of “ethnic density” could also be beneficial for healthcare outcomes for families living with a diagnosis of SCD. Facilitation, formation, and support for such groups (in person or online) could prove advantageous to increase feelings of parental competency and combat isolation in rural communities, or anywhere families find themselves with a diagnosis of SCD without local community support.

Geography may not be a significant barrier to care for patients with SCD if they have appropriate support within their community, such as the use of telemedicine, satellite campuses distributed throughout rural areas, programs to ensure proper prophylaxis and prevention, and contact with other patients and families who understand what it is like to live with SCD. Satellite campuses may prove to be of greater utility if flexibility in scheduling were improved to match the needs of the corresponding patient population.

Racial biases as they relate specifically to the patient with SCD still need to be addressed within the healthcare system. Evidence is especially strong for the lack of management of pain levels in these patients, which can be remedied by holding healthcare workers accountable for the language they use to describe these patients, as well as uncovering underlying biases within the system and individuals themselves. Additional training to educate healthcare workers on the pain and associated symptoms may help recognize and reconcile potential biases. Social support has a positive impact on patient outcomes, providing evidence for healthcare providers to take a more holistic approach to caring for these individuals. However, providers should focus not only on the physical ailments affecting these patients but also provide resources to aid in their psychological ones.

## Conclusions

This investigation aimed to examine the impact of race, SES, and geography on health-related outcomes in pediatric patients with SCD in the US from 2017 to 2022. Barriers to care for these patients are multifaceted, making it difficult to isolate and analyze the impact of individual variables. Further research is needed to assess the influence of each variable on this population, as well as the interplay between them. Isolation of these variables in the analysis is deemed necessary for evaluation. Objective definitions of these variables would allow for comparison between cohorts and studies, as well as the importance of each variable as it relates to outcomes. From the vantage point of the three domains this study investigated, the importance of race, SES, and geographical location specifically, it would help understand the importance of each variable in relation to the other. While many studies touched on the significance of relationships and social resources for these patients, a more thorough investigation should be performed regarding the benefits of support groups and support systems for the health outcomes of pediatric patients with SCD. Future investigations could evaluate barriers to care by considering the viewpoints of providers, other healthcare team members, peers without a diagnosis of SCD, and extended family members. Studies involving direct comparison to control groups of high SES in urban settings, such as those that occur with primarily white cystic fibrosis patients, may help unravel these variables from each other. The healthcare journey for patients with SCD proves to be just as complex as the disease itself, and closing these knowledge gaps is imperative in improving the care delivered to these children.
